# Preference and detrimental effects of high fat, sugar, and salt diet in wild‐caught Drosophila simulans are reversed by flight exercise

**DOI:** 10.1096/fba.2020-00079

**Published:** 2020-12-04

**Authors:** Alexander K. Murashov, Elena S. Pak, Chien‐Te Lin, Ilya N. Boykov, Katherine A. Buddo, Jordan Mar, Krishna M. Bhat, Peter Darrell Neufer

**Affiliations:** ^1^ Department of Physiology & East Carolina Diabetes and Obesity Institute East Carolina University Greenville NC USA; ^2^ Department of Molecular Medicine University of South Florida Tampa FL USA

**Keywords:** Drosophila simulans, exercise, food preference, mitochondrial efficiency, obesity, western diet

## Abstract

High saturated fat, sugar, and salt contents are a staple of a Western diet (WD), contributing to obesity, metabolic syndrome, and a plethora of other health risks. However, the combinatorial effects of these ingredients have not been fully evaluated. Here, using the wild‐caught Drosophila simulans, we show that a diet enriched with saturated fat, sugar, and salt is more detrimental than each ingredient separately, resulting in a significantly decreased lifespan, locomotor activity, sleep, reproductive function, and mitochondrial function. These detrimental effects were more pronounced in female than in male flies. Adding regular flight exercise to flies on the WD markedly negated the adverse effects of a WD. At the molecular level, the WD significantly increased levels of triglycerides and caused mitochondrial dysfunction, while exercise counterbalanced these effects. Interestingly, fruit flies developed a preference for the WD after pre‐exposure, which was averted by flight exercise. The results demonstrate that regular aerobic exercise can mitigate adverse dietary effects on fly mitochondrial function, physiology, and feeding behavior. Our data establish Drosophila simulans as a novel model of diet‐exercise interaction that bears a strong similarity to the pathophysiology of obesity and eating disorders in humans.

**Abbreviations**
CD: control diet
CDE: control diet and exercise
HFD: high‐fat diet
HSD: high sugar diet
SD: salt diet
WD: western diet
WDE: western diet and exercise

## 1 INTRODUCTION

The prevalence of obesity has expanded to pandemic proportions over the last few decades to all parts of the world and is strongly linked to the increased spread of Western lifestyle and Western diet (WD).^1^ The WD, notorious for its high fat and sugar content, predisposes individuals to obesity, metabolic syndrome, T2D (type 2 diabetes), cardiovascular diseases (CVD), inflammation, and behavioral disorders including bulimia and binge‐eating.^1^, ^2^, ^3^ High salt content is yet another hallmark of the WD, which contributes directly to high blood pressure, CVD, stroke, and renal diseases.^4^ There is growing evidence that high dietary sodium indirectly contributes to obesity through the relatively high fat and calorie content of salty foods and the fact that salty snacks are highly palatable.^5^ The combined adverse effect of excessive dietary fat, sugar, and salt is currently underestimated and rarely studied.

Recently, *Drosophila* has become a valuable model system for studying metabolic homeostasis and obesity‐related mechanisms.^6^ Metabolic regulation in *Drosophila* is highly conserved during evolution and shares important similarities to both mammalian energy metabolism and the target metabolic organs including heart, kidney (Malpighian tubules), liver, and adipose tissue (fat body), brain, and gastrointestinal tract.^7^ *Drosophila* insulin‐like peptides (DILPs) share structural and functional similarities with vertebrate insulin‐like growth factor and insulin.^8^ The response of fruit flies to a high‐fat diet (HFD) shows major similarities to mammalian obesity models with increased triglyceride and glucose levels, decreased stress tolerance, and reduced lifespan.^9^ Likewise, an HFD in *Drosophila* induces cardiac fat accumulation and cardiac dysfunction resembling phenotypic changes seen in human obesity.^10^ Transcriptomic analyses reveal HFD‐induced changes in the expression of genes in the metabolic pathways, cell signaling, motor function, and olfaction.^11^ Behaviorally, HFD impairs climbing ability and memory recall suggesting overall neurological decline.^12^ Thus, these recent studies highlight the suitability of the Drosophila model system for examining connections between high fat diet‐induced obesity and associated health risks.

Sugar is a common and natural ingredient of the Drosophila diet in nature as they feed on soft rotting fruits that are rich in sucrose, fructose, and glucose.^13^ These simple sugars provide calories for natural activity including high‐energy hovering flights.^14^ Under laboratory conditions, however, activity is restricted and fruit flies on a chronic high‐sugar diet (HSD) develop obesity, type 2 diabetes‐like pathophysiology, increased triglycerides, hyperglycemia, and lower taste responses to sweet stimuli, resulting in overeating.^15^ Furthermore, chronic HSD also leads to fibrosis‐like deterioration of heart function, insulin signaling defects, and a shorter life span.^16^

Salt is yet another fundamental nutrient that is required for many physiological processes in Drosophila.^17^ Fruit flies are excellent for studying the regulation of sodium levels as they are tolerant of high levels of dietary salt. Drosophila reared on salt‐rich diets demonstrate various alterations including changes in the secretion of Na^+^, K^+^, and diuretic factors,^18^ expression of sodium/halide symporter gene, and GLUT4/8‐like sugar transporter,^19^ increased carbohydrate metabolism, and sleep fragmentation.^20^ Interestingly, *Drosophila* responses to dietary NaCl bear strong similarities to mammalian behavior: low‐salt concentrations provide an attractive stimulus, whereas high‐salt concentrations are avoided.^21^

Likewise, a similar change between fly appetitive and aversive behavior has been reported for sucrose with reinforcement in a range from 0.25 to 2 M, and suppression of feeding at high concentrations (4 M).^22^ Matching observations in humans show that the taste preference for sugar follows an inverted U‐shaped curve^23^ as liking increases with added sweetness, reaches a so‐called “hedonic breakpoint”,^24^ and then declines when the product is perceived as too sweet. Moreover, a strong sensory synergy between sugar and fat appears to exist: the highest hedonic ratings in volunteers were obtained for sweetened light cream in comparison to sweetened skim milk and unsweetened heavy cream.^24^ However, sensory studies on salt and fat mixtures are very limited,^24^ and to the best of our knowledge, no studies have been performed on the combined impact of fat, sugar, and sodium in humans or any other organisms.

The findings from the present study document for the first time that a WD with a combination of high fat, sugar, and sodium is more detrimental to fruit fly health than each ingredient alone. The combination WD increased mortality rate, triglyceride content, and decreased functional capacity, the latter characterized by a decrease in mitochondrial oxidative phosphorylation (OXPHOS) efficiency and capacity in flight muscle. Strikingly, daily aerobic flight exercise completely or nearly completely mitigated all of the detrimental effects of the WD. Flies developed a preference for the WD despite its deleterious effects on physiology and health, mimicking behavior observed in humans and laboratory rodents. These results suggest that Drosophila can serve as an excellent model for studies on the molecular pathogenesis underlying obesity and diet‐induced metabolic and behavioral disorders in humans.

## 2 MATERIALS AND METHODS

### 2.1 Drosophila culture

Flies used in all experiments were derived from a colony established from six Drosophila simulans isofemale lines collected in 2018 in Greenville, NC. Flies were identified according to identification guide^25^ and maintained as an outbred population. The fly stock was maintained on the standard Bloomington Formulation diet (Nutri‐Fly® BF, Cat #: 66–112, Genesee Scientific Inc., San Diego, CA) in a climate‐controlled environment at 24°C under a 12 h light‐dark cycle and 70% humidity. All experiments were performed on age‐matched 3‐4‐day‐old flies. All flies, from the embryo stage, were raised on the standard Nutri‐Fly Bloomington diet (CD). Three to four‐day‐old flies were transferred to the WD, HFD, HSD, SD, or CD food, which were made based on standard Nutri‐Fly Bloomington diet with the following adjustments.

WD‐ 15% Nutiva USDA Certified Organic, non‐GMO, Red Palm Oil, 15% Sucrose, 0.1 M NaCl; HFD‐15% Nutiva USDA Certified Organic, non‐GMO, Red Palm Oil;

HSD‐ 15% Sucrose;

SD‐ 0.1 M NaCl;

CD‐ standard Nutri‐Fly Bloomington diet.

### 2.2 Behavioral experiments

The activity was measured in groups of five flies housed in narrow vials with food with 3–4 replicates per diet. Locomotor activity was measured using LAM25H locomotor activity monitors (TriKinetics Inc, Waltham, MA). To generate survival curves, survival was scored daily for each diet. Dead flies were counted at the same time of the day in groups of five flies housed in narrow vials. Survival was calculated as a percent of live flies for each diet group with 3–4 replicates. For flight exercise groups of sixty 3–4‐day‐old male flies were housed in 1‐gallon clear plastic drum fish bowls (Petco, San Diego, CA) strapped to a horizontal platform attached to a motor. The motor was controlled by two timers initiating three motor revolutions spaced 14 seconds apart every 5 min. Each revolution elevated the platform and then dropped it down triggering flies into flight. The exercise was performed daily for 7 h for 5 days. No mortalities or injuries associated with exercise were observed. The climbing assay was modified from the procedure described elsewhere.^26^ Climbing ability was measured in groups of 20 flies in three trials. The number of flies able to reach a 70 ml target line in a 100 ml glass cylinder was measured every 5 seconds during a 1‐minute trial. Respirometry was performed on groups of five flies housed in narrow vials with food with 3–4 replicates per diet and the experiment was repeated at least three times. The protocol essentially followed the procedure described previously^27^ to measure the amount of produced CO2. Briefly, the flies were quickly slowed down by cold metal block on wet ice and transferred to screw‐top 2 ml Eppendorf tubes, with soda lime pellets for CO2 absorption, attached to a long capillary submerged in a colored water. Afterwards, they were placed into sealed chambers and their CO2 production was measured during a 2 h period.

### 2.3 Triglycerides, glucose, and trehalose

Wet fly weights were taken immediately after freezing in liquid nitrogen. For dry weights, flies were killed in liquid nitrogen and then dried at 52ºC for 72 hours. Flies were individually weighed using Cahn C‐35 Ultra‐Microbalance (Thermo Fisher Scientific, Waltham, MA). The assays to measure triglycerides, glucose, and trehalose were carried out according to protocols modified from described previously.^28^ Briefly, five flies were rapidly homogenized in 0.5 ml of PBST (PBS with 0.1% Tween 20) using Bullet Blender (Next Advance, Inc., Troy, NY). The samples were centrifuged for 3 min at maximum speed at 4°C. Total proteins were measured using the Pierce Rapid Gold BCA Protein Assay Kit (A53225, Thermo Fisher Scientific). The assays for triglycerides and glucose were performed using incubations with Pointe Scientific Triglycerides Reagent (T7532120, Thermo Fisher Scientific) and Pointe Scientific Glucose Oxidase Reagent (23–666–288, Thermo Fisher Scientific), respectively. For trehalose measurements, samples were treated with porcine kidney trehalase (#T8778, MilliporeSigma, Burlington, MA) which converts trehalose into glucose, and thus total available glucose level was measured. Triglyceride, glucose, and trehalose contents were normalized to total protein levels per mean fly weight.

### 2.4 Mitochondrial respiration (JO2)

To assess the oxidative phosphorylation capacity of flies, mechanically permeabilized flight muscles were prepared as previously described.^29^ Briefly, flies were immobilized by cooling on cold block 0ºC and dissected by removing sequentially head, wings, legs, and abdomen. Flight muscle was made accessible by delicately breaking the thorax cuticle with 20‐gauge needles. The samples were weighed on Cahn C‐35 Ultra‐Microbalance (Thermo Fisher Scientific) and immediately transferred to the respirometer chamber filled with 0.9 ml of the respiration buffer. JO2 was measured as previously described^30^ with the following modification. Assay buffer was Buffer Z (105 mM K‐MES, 30 mM KCl, 10 mM KH2PO4, 5 mM MgCl2, 1 mM EGTA, 0.05% fatty acid‐free bovine serum albumin, pH 7.1)^31^ supplemented with 5 mM creatine (Cr). The experiment was carried out at 23°C (room temperature, assumed fly body temperature at rest.) in a 0.9 ml reaction volume with continuous stirring. Two protocols were performed to assess specific mitochondria complex linked JO2. Since proline was shown to be the dominate mitochondria substrate for insect’s flight muscle ^32^, ^33^, we assessed: 1) proline‐dependent state 4 respiration and 3 JO2; 2) complex I dependent state and 3 JO2 (proline, pyruvate, malate, ADP); 3) complex I plus II dependent state and 3 JO2 (+ succinate); 4) complex II dependent state and 3 JO2 (+ rotenone); 5) complex IV dependent state and 3 JO2 (+TMPD). In the second protocol, mitochondria were energized with 0.5 mM duroquinol (tetramethylhydroquinone) and 4 mM ADP for 10 min followed by the addition of 2.5 μM Antimycin A. Duroquinol donates electrons directly to complex III, while Antimycin A inhibits complex III electrons transport. This protocol allowed the assessment of complex III dependent state 3 JO2.

### 2.5 Mitochondrial respiratory control using the creatine kinase (CK) clamp

The CK clamp method and ΔGATP calculation were detailed in previously described^34^, ^35^ with the following modification. The experiment was carried out at 23°C in a 0.9 ml reaction volume with continuous stirring. Fly mitochondria were energized with 0.5 mM malate, 5 mM pyruvate, 5 mM proline, and 10 mMsuccinate. Phosphocreatin (PCr) was further titrated in the final concentrations of 2.5, 3.75, 7, 13, and 20 mM corresponded to ΔGATP of −12.88, −13.14, −13.46, −13.8, and −14.04 kcal/mol.

### 2.6 Western blot analysis

Tissues (dissected flight muscle or head) were extracted and homogenized in Extraction Buffer (20 mM Tris‐HCl pH 7.5, 5 mM magnesium acetate, 1 mM EDTA pH 8.0, 1 mM DTT, 1 mM PMSF in isopropanol, 1x PIC (Sigma Aldrich P2714), 0.5% Nonidet 40) for one minute on ice using a hand‐held homogenizer (Bel‐Art). The homogenate was then centrifuged at 13,000 rpm 5 minutes 4°C (Beckman). Volumes equivalent to one head/flight muscle were run through a 4%–12% Bis‐Tris precast gel (Invitrogen) and proteins were transferred to a 0.2 μM nitrocellulose membrane (Bio‐Rad). Membranes were then blocked in 25 ml of 1× TBST (Tris Buffered Saline 0.1% Tween‐20) +5% powdered milk and incubated in 5 ml of primary antibody solution. All secondary antibodies were diluted in 1x TBST +1% powdered milk. Membranes were then incubated in Immobilon™ Western Chemiluminescent HRP Substrate (MilliporeSigma) according to manufacturer's directions and imaged using an Amersham Imager 600 (GE Healthcare Life Sciences). Primary antibodies used include CoxIV (mouse, 1:2000 or 1:5000, Abcam), ATP5a (mouse, 1:20,000, Abcam), Actin (mouse, 1:2500, Abcam), Actin (rabbit, 1:2500, Abcam), Tubulin (rabbit, 1:5000, Abcam), Ref2p (rabbit, 1:800, Abcam), ChAT4B1 (mouse, 1:500, DSHB). Secondary antibodies used include horseradish peroxidase‐conjugated Donkey anti‐Mouse (1:10,000, Jackson Laboratories) or Goat anti‐Mouse (1:20,000, Jackson Laboratories) secondary antibodies.

### 2.7 Food‐preference

Two choice food preference tests were modified from the procedure described previously.^36^ Briefly, flies collected in groups of 25 males and 25 females were starved for 20 hr in wide vials with wetted filter paper. Colored food was prepared by mixing 130 ul of red or blue food coloring with 10 ml of food while it was liquid and hot. The red and blue foods were spotted as ~5 mm diameter round boluses (four red and four blue in alternating sequence) on the perimeter of the Petri dish (100 mm × 15 mm). The flies were allowed to feed for 120 min. The number of flies that were blue (NB), red (NR), or purple (NP) were counted. The preference indexes (PI) were calculated according to one of the following equations:

If the experimental diet of interest was mixed with the red dye, PI = (Nred + 0.5Npurple)/(Nred + Nblue + Npurple). If the experimental diet of interest was mixed with the blue dye, (Nblue + 0.5Npurple)/(Nblue + Nred + Npurple). PIs of 1.0 and −1.0 indicate complete preferences for one food option or the other. A PI of 0 indicates no bias between the two food alternatives.

### 2.8 Y‐maze

The experiments followed the procedure described previously with minor modifications.^37^ Groups of 25 males and 25 females were starved for 20 hr in wide vials with wetted filter paper. Starved flies were introduced into the “loading” chamber leading into Y‐splitter connected to “trap” vials with CD and WD food and allowed to choose between CD and WD vials for 6 hours in the dark. At the end of the experiment, the flies were frozen at −20°C and counted. The olfactory PI was calculated using the following formula: (number in the WD tube ‐ the number in the CD tube)/total number of loaded flies.

### 2.9 Statistics

Statistical analyses were perfomed using student t‐test, a one‐way ANOVA or a two‐way ANOVA, depending on data and variables, using GraphPad Prism version 8.00 for Windows, (GraphPad Software, San Diego, CA). Post hoc analyses were conducted using Tukey's test.

## 3 RESULTS

### 3.1 Western diet significantly affects mortality, activity, and reproduction in Drosophila

**Figure 1:**
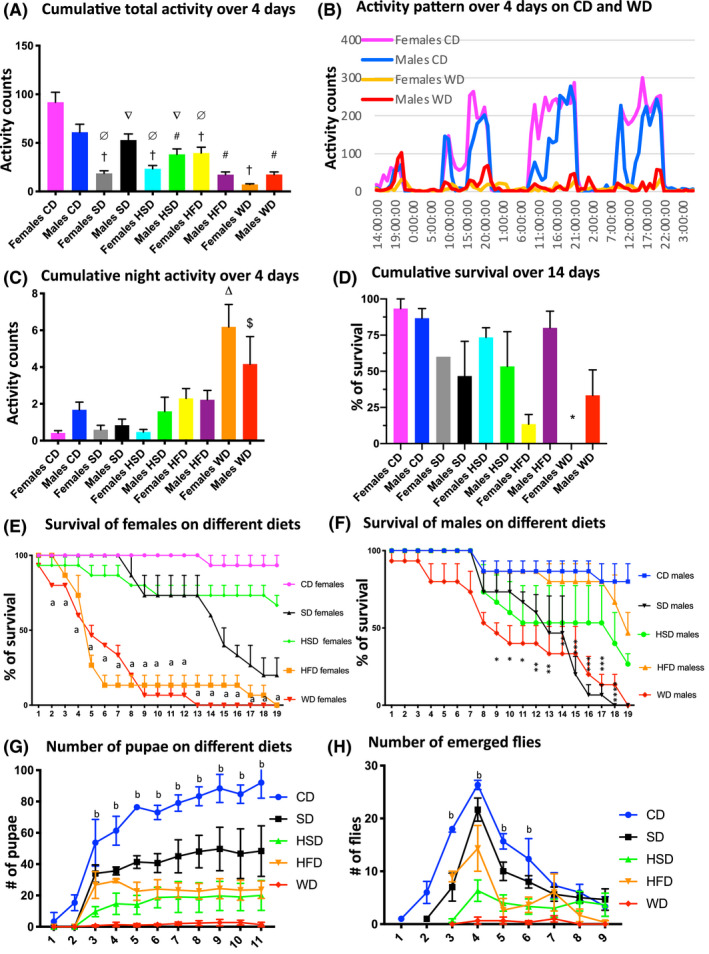
Effects of different diets on activity, survival, and development. (A): Total activity was recorded in a group of 5 flies in narrow vials over 4 days in LAM25H TriKinetics activity monitor (n = 9 replicates, 5 flies per replicate). Y‐axis ‐activity counts. Two‐way ANOVA. Tukey's multiple comparisons test. Significant differences between different diets and the CD: †‐ versus Control females (*p* < 0.0001), # ‐ versus Control males (*p* < 0.0001). Significant difference between different diets and WD: ∅ ‐ versus WD females (*p* < 0.01–0.0001), ∇ ‐ versus WD males (*p* < 0.05). (B): Representative graph of circadian activity pattern of females and males maintained on a 12‐h LD cycle (lights on at 9:00am) over 4 days. Note a decrease in activity on the WD during daytime and increase at nighttime. Locomotor activity is expressed as the mean number of beam breaks per hour. (C): Total activity during night (9 pm ‐ 9 am) on different diets. Two‐way ANOVA, Tukey's multiple comparisons test: △‐ significant difference between WD females and other female groups (*p* < 0.0001), $‐ significant difference between WD males and SD males (*p* < 0.05). (D): Cumulative survival over 14 days. Results are averages of nine replicates of 5 flies per vial. One‐way ANOVA. *‐ Significant difference between WD females and other female groups (*p* < 0.01–0.0001). (E, F): Survival of females on different diets (E). Two‐way ANOVA. a‐ significant difference between WD females and CD females (*p* < 0.0001). Survival of males on different diets (F). The number of flies that survived was determined for each cohort once per day. Two‐way ANOVA. *‐ significant difference between WD males and CD males (*p* < 0.05 – 0.0001). (G): Total number of pupae (mean ±s.e.m.) emerging from larvae sired by different fathers (CF, EF, WF, WEF). Two‐way ANOVA. b ‐ significant difference between WD and CD groups (*p* < 0.0001). (H): Cumulative number of eclosed adult flies (mean ±s.e.m.) sired by different fathers. Two‐way ANOVA. Tukey's multiple comparisons test: b ‐ significant difference between WD and CD groups (*p* < 0.0001). Error bars represent SEM. All experiments were repeated three times.

*D. simulans* collected from its natural habitat were used to investigate whether a combination of high fat, sugar, and sodium is more detrimental to health than each single ingredient. WD consisted of Bloomington diet with added NaCl to 0.1 M, sucrose to 15%, and palm oil to 15% by weight. Groups of female and male flies were exposed to WD, control diet (CD), HFD (15% palm oil), HSD (15% sucrose), or sodium diet (SD, 0.1 M NaCl). Fly locomotor activity was measured in TriKinetics LAM25H locomotor activity apparatus. ANOVA post hoc comparisons using Tukey's multiple comparisons test indicated a significant effect of diet condition (F (4,8) = 35.13, *p* < 0.0001). In female flies, all diets significantly decreased locomotor activity in comparison to CD; however, WD led to the most pronounced decrease in activity (Figure 1. A). In male flies, the activity was significantly reduced on WD, HSD, and HFD but not on SD. The most pronounced decrease in male activity was observed with WD and HFD. Overall, the activity of female flies on WD was markedly more suppressed than the activity of male flies. Interestingly, both female and male flies on WD exhibited a significant increase in activity at night (Figure 1B, C), suggesting disturbances in sleep patterns induced by the diet.

Analysis of the influence of the diets on survival showed a significant reduction in lifespan by salt, sugar and high fat supplementations (Figure 1E, F). Female flies were more sensitive to WD, surviving on average 13 days as compared to 19 days for male flies. Female fly lifespan was negatively impacted the most by the WD and HFD, while male flies were most susceptible to WD and SD. Taken together these results highlight sex differences in fly sensitivity to different diets, demonstrating that females are markedly more susceptible to high‐fat content.

The fertility of female flies on different diets was also studied by recording the number of pupae and newly emerged flies. WD produced the most detrimental effect on female reproduction and development (Figure 1G, H). No wandering larvae or pupae were observed on the walls of the vials with WD indicating impaired embryogenesis. Thus, only a few flies emerged from these vials. Development was also significantly delayed as new flies emerged on WD seven days later than control and were markedly smaller in size: WD females 2.014 ± 0.05 mm versus CD females 2.75 ± 0.0619 mm and WD males 1.9 ± 0.0377 mm versus CD males 2.36 ± 0.0421 mm (Diet effect, Two‐way ANOVA F (1, 6) = 181.7, *p* < 0.0001). While all diets inhibited embryo development, WD was the most toxic diet, while the SD had the least harmful effect on embryos. Thus, these data show that the combination of sodium, sugar, and fat is more detrimental to fecundity and development than each ingredient alone.

### 3.2 Exercise counterbalances the negative effects of the WD

**Figure 2:**
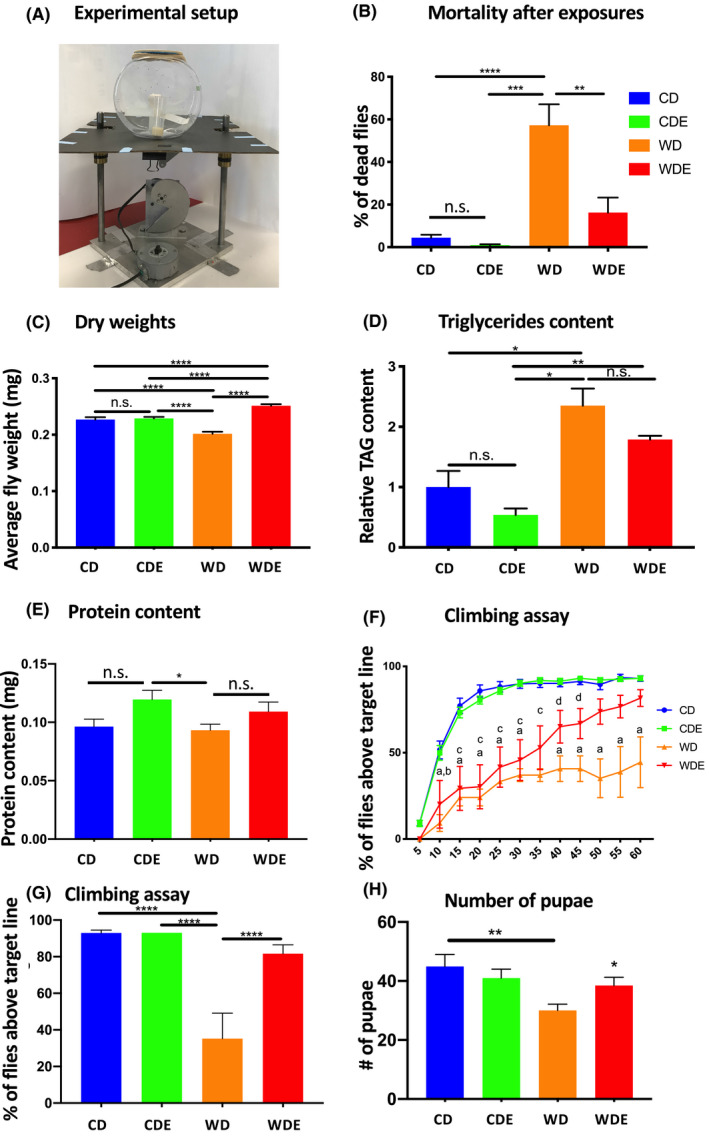
Exercise counterbalances the negative effect of the WD. (A): Experimental setup for flight exercise. Sixty 3‐4‐day‐old flies were housed in a 1‐gallon clear plastic fish drum attached to a horizontal platform. The motor controlled by two timers elevated the platform and then dropped it down triggering flies into flight. The platform was dropped three times every 5 min for 7 h for 5 days. No mortalities or injuries associated with exercise were observed. (B): Mortality of male flies subjected to WD and exercise combination. Blue color ‐ CD control flies (sedentary group on the CD); green color ‐ CDE exercise flies on CD; orange color – WD (western diet sedentary flies), red color – WDE (western diet +exercise flies). Total mortality was recorder at the end of 5‐day diet/exercise regimen. N = 10 trials, 60 flies/trial, error bars  = S.E.M. One‐way ANOVA treatment effect on mortality F (3, 15) = 16.70,*p* < 0.0001. ** ‐*p* < 0.01, *** ‐*p* < 0.001, **** ‐*p* < 0.0001. (C): Average fly dry weights. Flies were quickly killed in liquid after a 5‐day diet/exercise regimen and dried. Flies were individually weighed on an ultra‐micro balance. N = 3–4 trials, 50‐120 flies/trial, error bars  = S.E.M. One‐way ANOVA F (3, 334) = 35.47,*p* < 0.0001). (D): Relative triglyceride (TAG) content. TAG content was determined in whole body lysate of 5 flies and normalized to protein content per mean fly weight. N = 2 trials, 15 flies/trial, error bars  = S.E.M. One‐way ANOVA F (3, 7) = 12.17,*p* = 0.0036. (E): Protein content per mean dry fly weight. Welch's two‐tailed t‐test (*p* < 0.05, n = 8). (F): Climbing Assay. The percentage of flies having passed the threshold line is represented every 5 sec over the duration of the assay. N = 3–4 trials, 20 flies/trial, error bars  = S.E.M. Two‐way ANOVA showed a significant effect for time and diet/exercise factor (F (33, 319) = 5.641,*p* < 0.0001). Tukey's multiple comparisons test showed significant differences between groups: a‐ significant difference between WD and CD (*p* < 0.0001), b‐ significant difference between WDE and CD (*p* < 0.001), c‐ significant difference between WDE and CD (*p* < 0.01). (G): Climbing Assay. Percent of flies above the target line at 60 seconds. N = 3–4 trials, 20 flies/trial. Two‐way ANOVA F (3, 30) = 29.98*p* < 0.0001. (H): Total number of pupae (mean ±s.e.m.) emerging from larvae from different fathers. One‐way ANOVA F (3, 166) = 4.300,*p* = 0.0060, ** ‐*p* < 0.01. Unpaired t‐test: * ‐*p* = 0.0178 difference between WDE and WD (n = 44).

A growing body of evidence suggests that the harmful effects of excessive caloric intake and associated health ailments can be mitigated or even reversed by physical exercise in mammals.^38^ To determine whether the negative effects of WD can be prevented by exercise in the fruit fly model, 3 to 4‐day‐old adult male flies were randomly assigned to one of the following four groups: 1) control sedentary flies on CD; 2) sedentary flies on WD; 3) exercise flies on CD (CDE); 4) exercise flies on WD (WDE). These experiments were performed in male flies only because female flies reportedly do not show an adaptive response to exercise training.^39^ To stimulate repetitive flight exercise, 30 flies were housed in a 1‐gallon drum fishbowl attached to a platform (Figure 2A). The platform was connected to a motor that elevated the platform to 1 inch and then dropped it down. The drop impact was softened by rubber pads to prevent any injuries or stress on the flies. The timer attached to the motor initiated three drops, spaced 14 s apart every 5 min. This shaking triggered over 90% of flies into flying around the bowl. The exercise was performed during daylight from 8 am to 3 pm for 5 consecutive days. At the end of the 5‐day exercise regimen, mortality was the highest in the sedentary flies on WD (~40%) and lowest in the sedentary and exercise flies on CD (~1%). Mortality in WDE flies was significantly reduced in comparison to the WD group (~ 2‐fold), indicating that flight exercise markedly counterbalanced the negative effect of WD (Figure 2B).

To gain a better understanding of how the WD and exercise counteract each other, dry weight, triglyceride, and glucose levels were determined in the flies. WD significantly reduced the weight of the flies while exercise counterbalanced this effect (Figure 2C). One‐way ANOVA showed a significant effect of treatment on fly weight [F (3,334) = 35.47, *p* < 0.0001]. These results may seem counterintuitive because WD in vertebrates usually leads to weight gain and obesity. However, because the insect cytoskeleton does not allow the animal to expand its volume, the WD elicited a weight decrease due to a lower density of the fat tissue. The level of triglycerides in the whole‐body homogenate was the highest in WD flies and lowest in CDE animals (Figure 2D). Flight exercise significantly negated the impact of WD, reducing the levels of triglycerides in WDE flies compared with control (One‐way ANOVA F (3, 7) = 12.17, *p* = 0.0036). The glucose levels, while not reaching statistical significance, nevertheless showed a similar pattern with the highest level of glucose in WD, lowest in CDE, and WDE being close to control level (data not shown). Likewise, protein levels showed a clear trend to increase in exercise groups. While ANOVA did not show the statistical difference, Welch's two‐tailed t‐test indicated a significant difference between CDE and WD groups (Figure 2E). Taken together, these data show that flight exercise has a notable effect on fly physiology and counterbalances the effects of WD on mortality and adiposity in Drosophila.

Climbing ability is an important parameter of fly neuromuscular performance and locomotor function.^26^ Changes in climbing ability may be indicative of neurological, skeletal, and muscular abnormalities. Climbing ability was measured in groups of 20 flies in three trials. The number of flies able to reach a 70 ml target line in a 100 ml glass cylinder was measured every 5 seconds during a 1‐minute trial. The climbing test showed that while 70% of CD and CDE flies reached the target line by 15 sec, none of the WD flies were able to reach the target line. At the same time, flies in the WDE group were climbing significantly better, with the majority of the flies reaching the target line by 60 seconds (Figure 2F, G). These results demonstrated that flight exercise can counterbalance the detrimental effect of WD on climbing ability.

To evaluate the impact of the WD and exercise on male fertility, male flies after a 5‐day diet and exercise regimen were bred for two days with control virgin females. The number of pupae fathered by the flies on WD was significantly lower in comparison to control according to one‐way ANOVA (Figure 2H). Interestingly, exercise significantly counterbalanced the negative effect of WD according to the unpaired t‐test, bringing the number of pupae fathered by WDE closer to the control level. Taken together, these data demonstrate that the negative effect of WD on several aspects of fly physiology and behavior can be mitigated by flight exercise.

### 3.3 Impaired respiration and mitochondrial function with the WD

**Figure 3:**
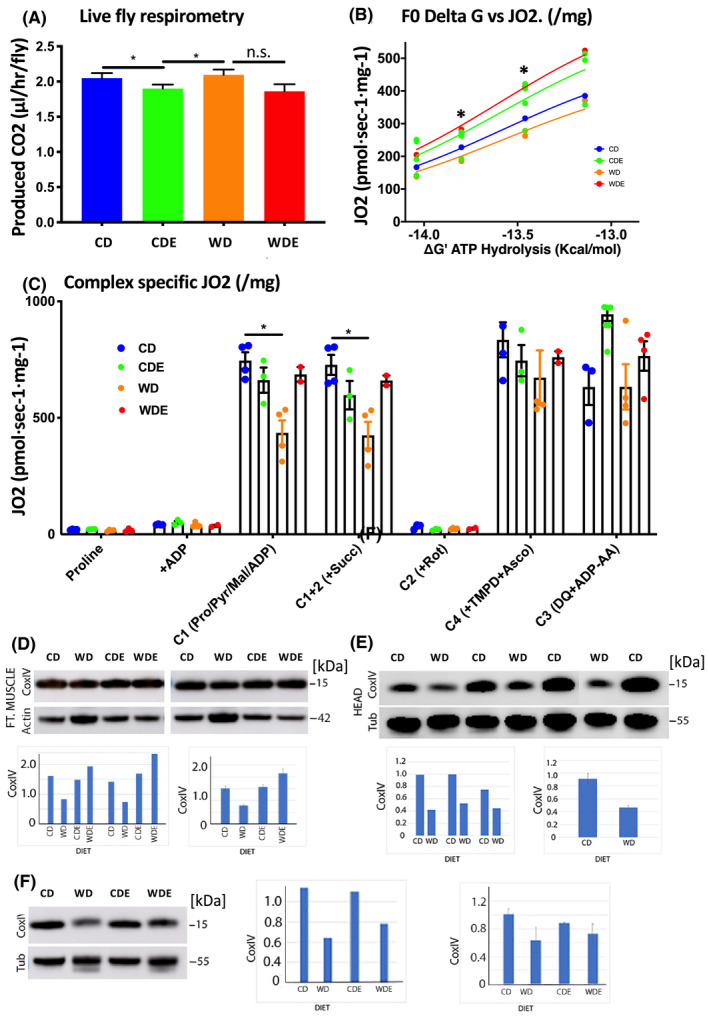
Effect of exercise and the WD on metabolism and mitochondrial respiration. (A): CO2 production in flies after exposure to the WD and exercise. Error bars indicate SEM from four individual experiments. Mann‐Whitney test (*‐*p* < 0.05, n = 27). (B): Respirometry on isolated and permeabilized flight muscles. ForceFlow Multi in O2 K: (mM) 0.5 malate/ 5 proline/ 5pyruvater/ 10 succinate/ 20U/ml of CK/ 2.5 PCr +10 ATP +PCr titer (3.75 + 7 + 13 + 20) +5 malonate/0.0025 AmA +2 ascobate +0.5 TMPD. N  =  3 trials, 20 flies/trial. Two‐way ANOVA, diet/exercise factor (F (3, 14) = 3.636,*p* = 0.0396). Tukey's multiple comparisons test: * ‐*p* < 0.05 WD versus WF. (C): Respirometry on permeabilized flight muscles. Analysis of complex specific JO2. Complex 124: 5 Proline +4 ADP +0.5 Mal/ 5 Pyr +10 Succ +0.002 Rot +5 Malonate/0.0025 AmA +2 Ascobate +0.5 TMPD. Complex 3: 4 ADP/0.5 Duroquinol +0.0025 AmA(mM). Analysis with Mixed‐effects model (REML) diet/exercise exposure factor F (18, 50) = 3.151,*p* = 0.0007. Tukey's multiple comparisons test: * ‐*p* < 0.05 CD versus WF. (D‐F): Effects of WD and exercise on CoxIV levels in the flight muscle. Western analysis of extracts from flight muscle (D) and head (E, F). ImageJ analysis was used to determine the intensity of bands in each lane. For flight muscle analysis, the levels of actin were measured for loading control. For head, tubulin levels were determined for loading control. The histograms showing the level of expression of CoxIV without the error bars represent the levels of protein in the Western blot. The histograms with the error bars represent averages of more than one Western blot run. For the flight muscle or the brain, the differences in CoxIV levels between CD and WD were statistically significant (*p* < 0.001; unpaired student t‐test). While there was no significant difference in CoxIV in flies fed with a CD with and without exercise in the flight muscle, the difference between WDE and WD flies was statistically significant (<0.001; student t‐test). In the brain, exercise did not have any beneficial effect on flies fed with CD, there was an exercise‐induced difference in flies fed with WD although it did not rise to statistical significance.

Diet‐induced obesity impairs energy metabolism by decreasing aerobic capacity, mitochondrial function, and increasing oxidative stress.^40^ Conversely, exercise can prevent or reverse these metabolically driven processes by accelerating energy expenditure, reducing adiposity, and improving insulin action.^41^ Since our above data show that the negative effects of the WD on fly physiology can be mitigated by exercise, we investigated whether bioenergetic processes are affected by WD and exercise in flies. Resting metabolic rate was determined by whole‐body respirometry over 2 hours in capillary tubes containing groups of five flies as described previously.^27^ One‐way ANOVA showed significant effect of diet and exercise (F ^397^ = 2.004, *p* = 0.1185) (Figure 3A). There were also significant differences between CD and CDE, and CDE and WD according to the Mann‐Whitney test (*p* < 0.05, *n* = 27). Thus, CO_2_ production was low in the exercise group (CDE) and high in the WD group, while the production of CO_2_ in the WDE group was closer to the control (Figure 3A). These results indicate that the CDE group became more energy‐efficient, while the WD group became more energy wasteful. Likewise, flight activity counterbalanced the negative effects of the WD possibly by increasing metabolic fitness.

ATP production derived through oxidative phosphorylation (OXPHOS) provides the energy needed to support flight activity. To assess the potential impact of the diet and exercise interventions on the OXPHOS capacity of flies, mechanically permeabilized flight muscles were prepared as previously described ^29^ and used to measure the mitochondrial respiratory capacity in single fruit flies by high‐resolution respirometry. To validate this model, we performed experiments to evaluate flight muscle permeabilization and the integrity of mitochondrial membranes to different substrates. Proline has been previously reported to be the dominant respiratory substrate in mitochondria isolated from the Drosophila flight muscle.^32^, ^33^ However, both basal and ADP‐stimulated oxygen consumption rates (*J*O_2_) were very low during respiration supported by proline or glutamate (not shown) with no difference detected between groups. Subsequent addition of pyruvate and malate increased ADP‐stimulated respiration by >10‐fold in all groups, suggesting that proline alone is insufficient to fully support mitochondrial OXPHOS in flies. Maximal *J*O_2_ during respiration supported by complex I substrates was reduced by 42% in flight muscle from WD as compared with control flies but was restored by the inclusion of exercise in the WDE group (Figure 3B, C). Similarly, *J*O_2_ values were also consistently higher over a large range of submaximal ADP‐stimulated respiration rates (i.e., clamped submaximal ΔG_ATP_ values) in WDE versus WD flight muscle, indicative of a compromise in OXPHOS efficiency with WD. Together these data suggest that complex I‐supported OXPHOS function is impaired in the flight muscle of flies within 5 days of starting a WD but is mitigated when WD is combined with exercise.

### 3.4 WD causes impairment of mitochondrial function in the brain and flight muscle

To begin to explore whether the WD may impact mitochondrial function in the fly flight muscle, the expression of CoxIV (cytochrome c oxidase subunit IV isoform), a key regulatory subunit of Complex IV, was determined in lysates prepared from the dissected flight muscle of flies. As shown in Figure 3D, CoxIV expression was significantly reduced in flies fed with the WD compared to the flies fed with CD (Two‐sample *t*‐test, *n* = 3, *p* = 0.00376863). Because CoxIV can serve as an index of mitochondrial content in the cells, it is possible that flies on the WD have a reduced number of mitochondria in the flight muscle. The CoxIV reduction suggests that the derangement of glucose/insulin homeostasis is transferred to the higher‐order organization of the respiratory chain and may represent another degree of mitochondrial damage by WD. We next analyzed if the levels of CoxIV improves with exercise. As shown in Figure 3D, indeed, exercise increased the levels of CoxIV in flight muscles of flies fed with the WD. The beneficial effects of exercise on CoxIV levels were much more pronounced in flies fed with WD than CD (Two‐sample *t*‐test, *n* = 3, *p* = 0.003). These results indicate that exercise reverses some of the deleterious effects of WD on flight muscle.

In addition to decreased mitochondrial function in peripheral tissues, obesity in humans is also associated with gradually developing pathology in the CNS, increasing the risk of cognitive decline and neurological disorders including Alzheimer's disease.^42^ Since flies fed with WD showed certain behavioral deficits, we sought to examine if the levels of CoxIV is affected in the brain. Head extracts were prepared from flies fed with CD, WD, and also those who were subjected to exercise. As shown in Figure 3E, flies fed with the WD had a significant reduction in the levels of CoxIV (Two‐sample *t*‐test, *n* = 3, *p* = 0.02). While the exercise did not enhance CoxIV levels in CD fed flies, as in flight muscles, exercise increased the levels of CoxIV in the head extracts of flies fed with WD (Figure 3F). These results are consistent with the other results that showed the deleterious effects of WD on mitochondria.

### 3.5 Flies show a preference for the WD compared to CD with the sex differences

**Figure 4:**
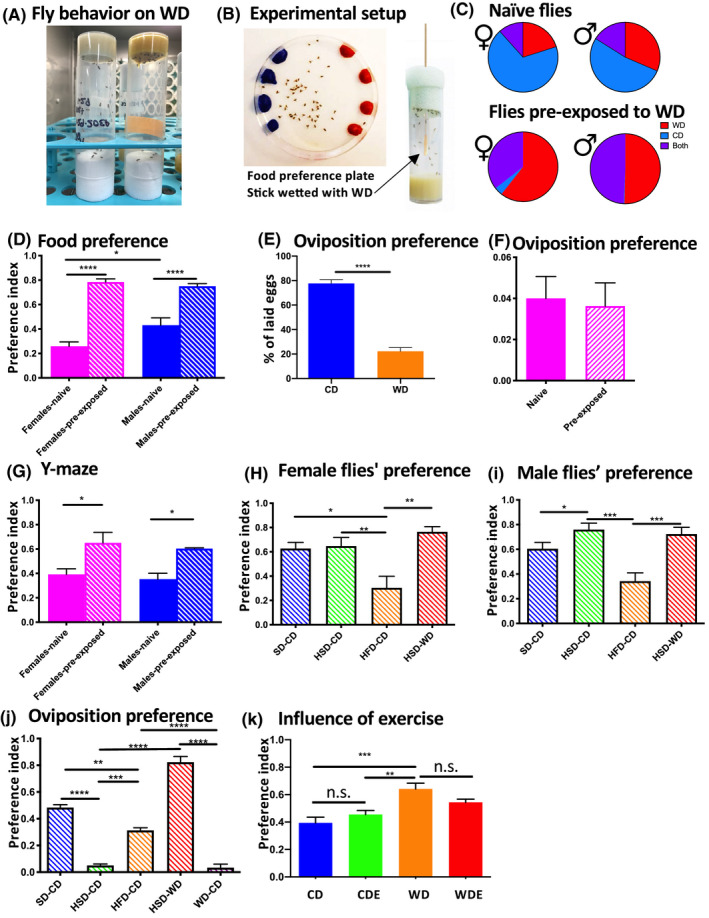
Food and oviposition preference for different diets. (A): Example of fly behavior on the WD. Flies concentrate on the WD substrate most of the time even when vials are kept inverted. Left vial with CD, a right vial with WD. (B): Experimental setup. Right: Two‐choice colorant feeding assay. Flies can choose to feed on CD or WD substrates colored with different food dyes (which are switched in different trials). Feeding preference is determined by the abdominal coloration of flies after 120 min. Left: Illustration of pre‐exposure. WD‐wetted stick was introduced into a vial with control food for 5 hours prior food preference test. (C‐D): Food preference of naïve and pre‐exposed flies in the two‐colored assay. Pie diagrams illustrating food preference in two‐colored assays (C). Top circles illustrate that female naïve flies have a stronger preference for CD. Top left‐naïve females, top right‐naive males. The bottom circles illustrate that after pre‐exposer flies prefer WD. Bottom left‐pre‐exposed females, bottom right pre‐exposed males. Mean preference index of naïve and pre‐exposed flies in a two‐choice assay for WD versus CD (D). N = 3–4 trials, 50 flies/trial, error bars  = S.E.M. One‐way ANOVA, there is a difference in the mean values (*p* < 0.0001). Pairwise comparisons using Tukey's multiple comparisons test: * ‐*p* < 0.05 **** ‐*p* < 0.0001. (E‐F): Oviposition preference. Oviposition preference in naïve female flies WD versus CD (E). Y‐axis: % of laid eggs on CD or WD with the total number of eggs laid on Petri dish taken as 100. N = 5 trials, 50 flies/trial, t‐test: **** ‐*p* < 0.0001. Mean oviposition preference index to WD versus CD is not affected by pre‐exposure to WD in females (F). (G) Mean preference index for Y‐maze. N = 3–4 trials, 50 flies/trial, error bars  = S.E.M. One‐way ANOVA (F (3, 12)*p* = 0.0044). A pairwise comparison was performed using Tukey's multiple comparisons test. (H‐J): Preference for different diets. N = 3–4 trials, 50 flies/trial. Mean preference indexes for different diets in females (H). One‐way ANOVA (F (3, 19) = 8.509*p* = 0.0009). A pairwise comparison was performed using Tukey's multiple comparisons test. Mean preference indexes for different diet in males (I). One‐way ANOVA (F (3, 20) = 10.98*p* = 0.0002). Tukey's multiple comparisons test. Mean oviposition preference indexes for different diets (J). One‐way ANOVA (" (4, 12) = 140.2*p* < 0.0001). Tukey's multiple comparisons test. (K): Influence of exercise on food preference. Mean preference indexes for WD versus CD after diet and exercise exposures. N = 3 trials, 30 flies/trial, error bars  = S.E.M. One‐way ANOVA (F (3, 24) = 8.979*p* = 0.0004). Tukey's multiple comparisons test.

Preference for energy‐dense foods is an important contributor to the current obesity epidemic.^43^ Both genetic, non‐genetic factors and acquired traits influence food preference through changes in gustatory and olfactory reception, feeding motivation, and appetitive and consummatory behaviors.^44^, ^45^ Flies exposed to WD demonstrated a lack of locomotor activity visible to the naked eye as the flies exposed to the WD spent most of the time sitting on the food even with the tubes inverted upside down (Figure 3A). Animals and humans learn about their likes and dislikes for food through tasting, feeling, smelling, and seeing. Experimental studies with children and adults demonstrate that pre‐exposure to food or drink often increases favorability.^46^ Thus, we hypothesized that fly behavior might indicate that flies have developed a preference for WD. A simple initial approach to verify this hypothesis is to test whether the consumption of energy‐dense food would lead to alterations in food choices. Therefore, in the next experiment, food preference behavior in naïve flies and those pre‐exposed to WD was studied using a two‐way choice assay modified from a previously described procedure.^36^, ^47^ Three to four‐days‐old naïve flies were starved for 20 hours and then allowed to choose for 120 min in the dark between CD and WD food boluses mixed with blue or red food dye (Figure 4B). To study whether pre‐exposure influences food preference in *Drosophila*, a stick wetted with WD was introduced into vials with naïve flies for 5 hours (Figure 4B). After a subsequent 20‐hour starvation period, the flies were then offered to choose between CD and WD (i.e., two choice assay). Abdomen color was then used to calculate the preference index. Initially, naïve males and females preferred consuming CD over WD food by ~1.7‐fold and 3.0‐fold, respectively. The preference for the CD in naïve flies was significantly more pronounced in females (Figure 4C, D). Surprisingly, a single pre‐exposure to WD significantly increased the preference for WD food in both males and females (Figure 4D). Females strongly avoided laying eggs on WD (Figure 4E), and the pre‐exposure to WD did not change female oviposition preference for CD (Figure 4F), suggesting that food preference and egg‐laying preference are regulated by different mechanisms.

To determine if pre‐exposure to WD affects appetitive behavior, flies were subjected to a Y‐maze test using a modified procedure.^37^ Starved flies were released into the “loading” chamber leading into Y‐splitter connected to “trap” vials with CD and WD food. Narrow pipette tips leading to “trap” vials prevented flies from returning once the arm choice was made. The flies could choose between CD and WD vials for 6 hours in the dark. The results revealed that 33% of the naïve flies chose WD, whereas 65% of the pre‐exposed flies preferred WD (Figure 4G). These results demonstrate that pre‐exposure to WD significantly changes food‐seeking behavior and food choices.

To determine whether food choice in naïve flies was driven by a single ingredient in the WD (i.e., high fat, sugar, or salt content), two‐way choice assays with foods containing only one ingredient were compared as follows: SD versus CD, HSD versus CD, HFD versus CD, and HSD versus WD. Both female male and male flies preferred SD and HSD over CD but avoided HFD food. When HSD was pitted against WD both females and males chose HSD (Figure 4H, I). For oviposition, females rejected HSD, HFD, and WD substrates (Figure 4J). Therefore, the pre‐exposed flies possibly developed a preference for WD because of high salt and sugar content and despite present palm oil. Conversely, high sugar and fat content were possibly responsible for the avoidance of WD as an oviposition substrate.

We then asked whether exercise may affect food preference in flies. CD, CDE, WD, and WDE groups of flies were tested after diet/exercise regimen for food preference. Not surprisingly, flies on the WD significantly preferred WD in comparison to CD and CDE (Figure 4K) However, what is interesting here is that WD‐exercise combination in the WDE group produced a trend to lower preference for WD (WEF vs. WF, *t*‐test, *p* = 0.1158), indicating that exercise might affect food choice in flies.

## 4 DISCUSSION

Using the Drosophila system, the present study demonstrates that a WD enriched with a combination of saturated fat, sugar, and salt is more detrimental to lifespan, locomotor activity, and reproductive function than each ingredient alone. The detrimental effect of WD and decreased mitochondrial respiratory function can be negated or even reversed by daily aerobic exercise. Remarkably, the WD is preferred over CD despite its harmful health outcomes of WD, suggesting that these preferences may be hard‐wired based on caloric richness and/or taste. These results may have implications for the human diet and health given that obesity is a major risk factor for all types of diseases.^48^, ^49^, ^50^, ^51^, ^52^

We sought to analyze flies that were caught from their natural habitat (wild). This population consists of genetically heterogeneous individuals whose genetic, physiological, and bioenergetic repertoire has been shaped by the “real world” environment.^53^ It has been suggested previously that long‐term laboratory strains, acclimated to living in benign and nutrient‐rich conditions, may exhibit altered physiological reactions.^54^ Observations in flies suggest that long‐term laboratory cultures maintained on short, discrete‐generation cycles are likely to have high fecundity, but low lifespan and resistance to starvation and desiccation compared to wild populations.^55^ Analogous comparisons in laboratory rodents revealed lower fecundity in the wild‐derived mice which ate less food, grew more slowly, and became sexually mature later than laboratory animals.^56^ Consequently, laboratory animals may respond to metabolic challenges via physiological mechanisms different from those that would be seen in wild populations.^57^, ^58^ Given the above evidence for variation between wild‐caught and laboratory animals, we employed wild‐caught *Drosophila simulans* to examine innate phenotypic traits and associated metabolic responses to WD.

### 4.1 Negative effects of the WD are due to the combination of high salt, sugar, and fat

Although human studies demonstrate that energy‐dense foods rich in fat, sugar, and salt are more palatable and preferred over plant‐based diets, the molecular and cellular mechanisms by which combination of these ingredients impacts physiology and bioenergetics are not well understood.^59^ Furthermore, while the palatability of energy‐dense foods is recognized as an important contributor to the obesity epidemic, very little is known as to how the combination of fat, sugar, and salt influences complex feeding behaviors. Previous studies have shown that feeding a high‐fat diet (adding coconut oil, for example) to flies ^60^, ^61^ or high‐sugar supplementations ^62^, ^63^ to model T2D and insulin resistance, elicits an obese phenotype with decreased lifespan and fecundity, elevated triglycerides and reduced cardiac function.^8^, ^15^, ^64^, ^65^, ^66^ The effect of sodium chloride in Drosophila has mostly been studied in the context of salt stress and taste perception, with concentrations above 2% shown to negatively impact fly survival and concentrations higher than 4% proving fatal to both larvae and adults.^17^, ^18^, ^19^, ^21^, ^67^ Interestingly, we observed significant sexual dimorphism in responses of flies to different diets. Female flies were especially susceptible to the WD and HFD surviving no longer than 13 days, whereas male flies survived on the WD for up to 19 days and on the HFD up to 25 days. While these mortality data are consistent with previous findings showing increased mortality on the HFD (10), HSD (16), and high‐salt (19) diets, our data show that a combination of high‐fat, sugar, and salt is more detrimental than each ingredient alone. The over 40% mortality of WD‐fed flies could suggest either potential toxicity of the combination diet or dehydration due to higher sugar and salt content. In additional experiments, we have provided water *ad libitum* to the flies and found that it did not affect survival on the WD (data not shown).

Additionally, nighttime activity in the WD‐fed flies was significantly increased in both sexes, which is indicative of sleep disturbance, whereas daytime activity was significantly reduced, and this was more pronounced in females. While these effects may reflect a cumulative burden on metabolism, what mediates the sex difference in some responses is not clear.

### 4.2 Exercise reverses the WD‐mediated adverse effect on metabolic outcomes

Previous studies have demonstrated that regular aerobic exercise can help prevent and treat various obesity‐associated conditions including diabetes,^68^ CVD,^69^ and mental health issues.^70^ Drosophila could be used to model the effects of exercise on metabolism.^71^, ^72^ We used flight‐exercise in our work since flight in insects is a high‐energy aerobic exercise that involves the highest mass‐specific rates of aerobic metabolism compared to other forms of physical activity.^73^, ^74^While hovering, Drosophila wings reach flapping frequencies of up to 1000 Hz with an associated increase in aerobic metabolism rates of up to 100‐fold.^74^, ^75^ Our study employed a modified protocol from previously reported method for inducing flight exercise,^76^ so that no injuries or deaths resulted from the exercise, as were reported for other types of exercise protocols, such as RING or Power Tower ^77^ (Figure 2A). Our data show that flight exercise reduced WD‐induced mortality by ~2‐fold (Figure 2B). Flight exercise also significantly improved the performance of the WD flies in a climbing test as well as reduced whole‐body triglycerides to the control level. Taken together, these results demonstrated significant benefits of aerobic exercise in our fruit fly model as well.

A potential primary mechanism by which aerobic exercise serves as an effective therapeutic intervention may simply be through the restoration and/or regulation of bioenergetic balance.^40^, ^78^ The simplest potential explanation is that exercise counterbalanced the negative effects of the WD by virtue of the additional energy expended during exercise. However, endogenous regulatory mechanisms also appear to be invoked which initially may seem counterintuitive. For example, resting metabolic rates were lowest in exercised animals and highest in sedentary animals on the WD, indicating that exercise makes flies more energy efficient while the WD makes sedentary flies more energy wasteful. Importantly, when flight exercise was combined with WD, the decrease in energy efficiency was prevented. In situ analysis of mitochondrial function in permeabilized flight muscle also revealed that a WD decreases mitochondrial OXPHOS capacity and efficiency, both of which are prevented when the WD is combined with exercise. The implication is that metabolic efficiency decreases when energy balance is positive, but transitions to maximizing energy efficiency when energy demand is high. Taken together, this points to the presence of internal mechanisms regulating the efficiency of mitochondrial bioenergetics, the nature of which is not yet known.

### 4.3 A preference for the WD may be hard‐wired

The cause and effect relationship between food choices and obesity can be addressed by studying food preference behavior in flies that are naïve and pre‐exposed to the WD. The experiments showed that naïve males and females preferred consuming the CD and avoided the WD as both food and oviposition substrate. Both the two‐way choice test and the Y‐maze test for appetitive behavior found that the majority of naïve flies choose the CD, whereas, the pre‐exposed flies choose the WD, demonstrating that pre‐exposure to the WD significantly changes food‐seeking behavior and food choices. A two‐way choice assay for single ingredients also found that naïve flies prefer food containing salt and sugar but avoid food with palm oil. These data argue that high salt and sugar content contributed to the palatability of the WD despite palm oil content. Interestingly, the preference for the WD in male flies was decreased by exercise. This is supported by recent observations that exercise training in human subjects leads to healthier dietary preferences^79^ and reduces overeating and reward for high‐fat food.^80^

One of the limitations of this study is that it did not measure the amount of food flies consumed while on the different diets. It would be interesting to investigate if pre‐exposure to the WD changes food motivation or induces hyperphagia. Future research needs to examine more closely the links between different diets and appetitive and addictive behaviors.

Finally, it would be interesting to do some direct comparisons to similar experiments perfomed in Drosophila melanogaster. Our preliminary observations indicate that naïve *Drosophila melanogaster* flies have a much higher preference for the WD and significantly lower mortality in comparison to *Drosophila simulans*(data not shown) suggesting that observed effects could be either species‐specific or due to different physiology of the wild‐caught flies.

### 4.4 Conclusion

Overall, this study shows, to our knowledge for the first time, that a combination of high fat, sugar, and sodium content is more harmful to Drosophila than each ingredient alone. Moreover, the deleterious effects of a WD are counterbalanced and reversed by daily flight exercise demonstrating strong applicability of the Drosophila model for studying not only obesity‐related mechanisms but also cellular and physiological mechanisms of exercise‐induced benefits. In addition, the acquired preference for the WD, despite the associated negative health impact, suggests that the reward experience in flies may be a model for higher‐order food‐seeking behavior and motivational control. The findings also demonstrate that *Drosophila simulans* is an excellent model system for studying the cellular and physiological underpinnings of obesity‐related disease processes as well as the mechanisms underlying exercise‐induced health benefits.

## CONFLICT OF INTEREST

The authors declare no competing financial interests.

## AUTHOR CONTRIBUTIONS

AKM designed the research. AKM, ESP, CTL, INB, KAB, JM performed the experiments. AKM, ESP, CTL, JM, KMB, and PDN analyzed the data. AKM wrote the manuscript. KMB and PDN revised the manuscript.
